# Casting Skin Dressing Containing Extractions of the Organic Part of Marine Sponges for Wound Healing

**DOI:** 10.1021/acsabm.4c01497

**Published:** 2024-12-20

**Authors:** Amanda de Souza, Cintia C. S. Martignago, Lívia Assis, Fernanda Vieira Botelho Delpupo, Marcelo Assis, Karolyne S. J. Sousa, Lais Caroline Souza e Silva, Laura O. Líbero, Flavia de Oliveira, Ana Claudia Muniz Renno

**Affiliations:** † Department of Biosciences, Federal University of São Paulo (UNIFESP), Lab 342, 136 Silva Jardim Street, Santos, SP 11015020, Brazil; ‡ Scientific Institute and Technological Department, University Brazil, São Paulo-Itaquera, SP 04021-001,Brazil; § CDMF - Department of Chemistry, Federal University of São Carlos (UFSCar), Washington Luís Road, São Carlos, SP 13565-905, Brazil

**Keywords:** *Chondrilla caribensis*, spongin-like collagen, chronic wounds, casting method

## Abstract

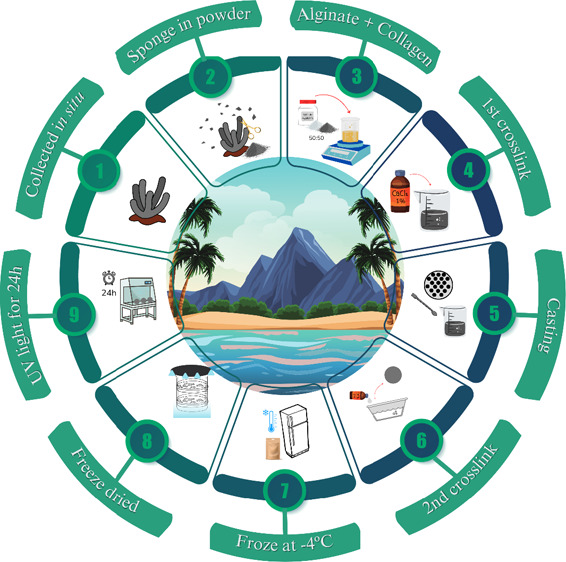

Skin wounds are extremely frequent injuries related to many etiologies. They are a burden on healthcare systems worldwide. Skin dressings are the most popular therapy, and collagen is the most commonly used biomaterial, although new sources of collagen have been studied, especially spongin-like from marine sponges (SPG), as a promising source due to a similar composition to vertebrates and the ability to function as a cell-matrix adhesion framework. Despite evidence showing the positive effects of SPG for tissue healing, the effects of skin dressings manufactured are still limited. In this context, this study aimed at investigating the effects of collagen skin dressings in an experimental model of skin wounds in rats. For this purpose, SEM, FTIR, cell viability, morphological and morphometric aspects, collagen deposition, and immunostaining of TGF-β and FGF were evaluated. The results demonstrated micro- and macropores on the rough surface, peak characteristics of collagen, and no cytotoxicity for the skin dressing. Also, the control group (CG) after 5 and 10 days exhibited an intense inflammatory process and the presence of granulation tissue, while the treated group (TG) exhibited re-epithelialization after 10 days. The evaluation of granulation tissue and neoepithelial length had an intragroup statistical difference (*p* = 0.0216) and no intergroup difference. Birefringence demonstrated an organized mesh arranged in a network pattern, presenting type I and type III collagen fibers in all groups. Moreover, in the morphometric evaluation, there were no statistical differences in intergroups or time points for the different types of collagen evaluated. In conclusion, these findings may indicate that the dressing has not exacerbated the inflammatory process and may allow faster healing. However, further studies using a critical wound healing injury model should be used, associated with longer experimental periods of evaluation, to further investigate the effects of these promising therapeutic approaches throughout the skin repair process.

## 1 Introduction

Skin wounds are extremely frequent injuries and are related to many etiologies, such as physicochemical and thermal damages, metabolic diseases such as diabetes, comprising the tissue leading to necrosis, pain episodes, a marked decline in quality of life, and even mortality of the patient.^1,2^ It is estimated that the costs associated with the treatment of severe and chronic wounds in the US affect approximately 5.7 million people and involve around USD 50 billion annually in treatments.^3,4^

In the therapeutic clinical setting, treatments primarily rely on surgeries, skin graft implantation, and the use of anti-inflammatory and analgesic drugs.^1,5,6^ Among these strategies, skin dressings play a pivotal role in protecting wounds, managing exudates, and preventing infections. Despite their importance, there remains a persistent demand for innovative dressings with enhanced biological properties to further optimize wound healing and improve treatment outcomes.^7−9^ Recent advancements in manufacturing techniques have expanded the potential of wound dressings, particularly through approaches such as casting, electrospinning, and 3D printing.^10,11^ Zhang et al.^12^ reviewed the application of electrospinning using alginate as a base material for nanofibers designed as drug release systems. Their findings highlighted the feasibility of this technique but underscored the need for further exploration of its functionality with diverse materials and methods, including advanced molecular simulations. Wang et al.^13^ developed a trilayer Janus fiber wound dressing composed of polyvinylpyrrolidone, berberine hydrochloride, cellulose acetate, and aloin. This dressing demonstrated biocompatibility, noncytotoxicity, and significant antibacterial properties, showcasing the potential of multilayer designs for advanced wound care applications. These studies illustrate promising avenues for integrating innovative materials and manufacturing techniques to create next-generation skin dressings with superior therapeutic outcomes.

Another critical factor in the success of wound treatment using skin dressings is the choice of raw material used in their manufacture. Among the various options, collagen has emerged as one of the most promising materials for this purpose.^14^ Collagen-based skin dressings, when applied to a wound site, serve as a protective barrier against infections while simultaneously integrating with the tissue. This dual action helps attract cells involved in regeneration, thereby promoting and accelerating the healing process.^7−9^ Collagen (Col) is the most commonly used biomaterial for these applications and can be extracted from the tissues of various species, primarily from the skin and bones of bovine and porcine.^15^ However, there are several drawbacks to using collagen obtained from mammals, including the possibility of disease transmission (such as bovine spongiform encephalopathy and foot-and-mouth disease) and certain cultural or religious prohibitions.^15^ To address these concerns, alternative sources of collagen have been explored, particularly from marine species. These include crustaceans, sea anemones, prawns, jellyfish, fish skin, and marine sponges, which offer a safer and more ethically acceptable means of collagen extraction.^8,15−17^ This shift toward marine-derived collagen highlights ongoing efforts to identify sustainable and versatile biomaterials for advanced wound care solutions.

Among these, marine sponges (phylum *Porifera*) have gained significant attention as a promising and ethically acceptable option for collagen extraction^18,19^. They are sessile animals, considered primitive in the animal kingdom and basis of the trophic chain.^20^ Their body structures are composed of many bioactive components,^21,22^ specially collagen (also known as spongin (SPG) or spongin-like collagen).^23,24^ SPG has been considered a natural compound for tissue bioregeneration due to the similarity to vertebrates Col. Also, it has the ability to function as a cell-matrix adhesion framework.^15,25,26^ Furthermore, SPG is capable of supporting human skin cell growth because of its biocompatibility.^16,27^ Among the SPG mechanical and chemical properties, the lower viscosity and water solubility, as well as the proportions of proline compared to mammalian Col, could be highlighted, which makes it more suitable for tissue engineering applications.^28^ For example, Pozzolini et al.^19^ manufactured a skin membrane for treating burns using SPG from marine sponges (*C. reniformis*) and observed that this material showed good mechanical properties, antioxidant activity, and biocompatibility with both fibroblast and keratinocyte cell cultures. Sales et al.^29^ investigated the association of a skin dressing manufactured with SPG from marine sponges and photobiomodulation in the process of skin injury healing and observed that treated animals presented re-epithelialization and the presence of granulation tissue at the area of the injury, stating that the associated treatments showed favorable biological effects on the skin repair process.

Despite evidence showing the positive effects of SPG for tissue healing, the effects of skin dressings manufactured with this raw material present limited knowledge. It is worthwhile to highlight that the development of optimal protocols for treating skin wounds is still ongoing, with an emphasis on achieving a therapeutic intervention that is faster, less expensive, and more effective. Considering this information, this study aimed to investigate the effects of a collagen skin dressing containing an extract of the organic part of marine sponge (SPG) in an experimental model of skin wounds in rats. For this purpose, we evaluated the morphological and morphometric aspects, collagen deposition, as well as the immunostaining of TGF-β and FGF.

## 2 Material and Methods

**Figure 1:**
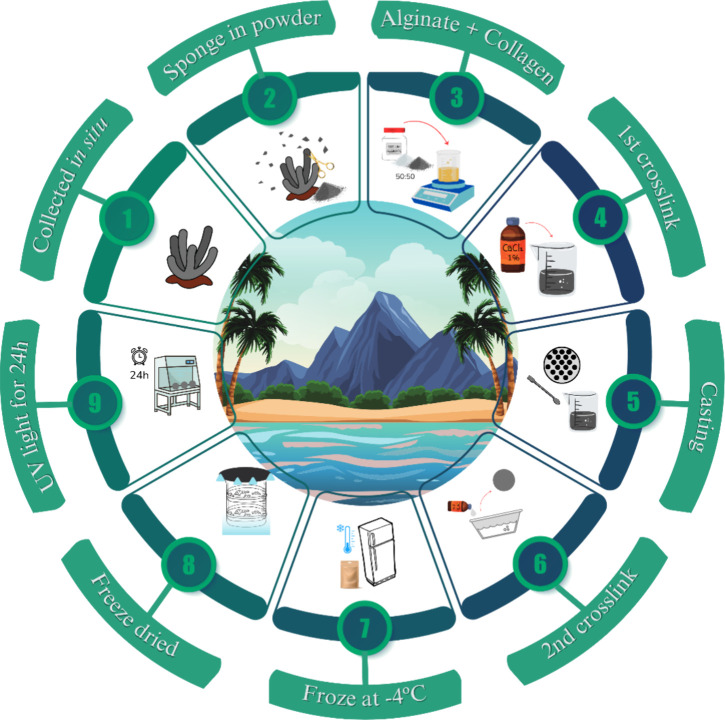
Schematic process of collection, SPG extraction, and SPG skin dressing manufacture.

The schematic process of collection, SPG extraction, and SPG skin dressing manufacture is briefly illustrated in Figure 1.

These processes, along with their implications for the development of advanced wound care materials, will be discussed in detail in the subsequent sections.

### 2.1 Marine Sponges

The marine sponge *Chondrilla caribensis* was used for SPG extraction after approval of the Brazilian National System for the Management of Genetic Heritage and Associated Traditional Knowledge (SISGEN–A21ED79) and the Biodiversity Authorization and Information System (SISBIO–28917–1). The animals were collected in situ at Praia Grande (23°49′23.76″S, 45°25′01.79″W, São Sebastião, Brazil), and the samples were briefly washed and transported in seawater to the laboratory. The pretreatment consisted of removing debris, cutting it into small pieces, and stirring the samples in distilled water for 2 h. In sequence, the samples were frozen at −20 °C, freeze-dried, and stored before undergoing collagen extraction.

#### 2.1.1 Marine Collagen Extraction

SPG extraction was performed according to Araújo et al.^18^ Briefly, the marine sponge *Chondrilla caribensis* , which was previously cleaned and stored at −20 °C, was cryogenically milled (Retsch Mixer Mill, MM400, Haan, Germany) for three cycles at 30 Hz for 2 min. The powder obtained was solubilized with deionized water. The solution was mixed in a vortex (AV-2, GEHAKA, Brazil) at room temperature for 15 min at 2500 rpm. Next, centrifugation was performed for 10 min at 5 °C and 5000 rpm, with the supernatant collected, followed by freezing the SPG at −20 °C, freeze-dried, and stored.

### 2.2 SPG Skin Dressing Manufacturing

The SPG skin dressings were manufactured through the casting method. Initially, 4 g of sodium alginate (ALG; CAS 9005–38–3, Sigma-Aldrich, St. Louis, MO, USA) and 4 g of SPG were individually weighted on an analytical balance (BEL Engineering; model: M214-AIH; Monza (MB); Italy) to achieve a ratio of 1:1. Here, sodium alginate had the purpose of the matrix for the SPG skin dressing solution. After that, sodium alginate was diluted in 100 mL of distilled water under heating up to 60 °C with constant stirring, and the solution was left at room temperature to cool to 20 °C. The next step was the addition of SPG in powder with constant stirring until a homogeneous solution could be obtained. After homogenization, the solution was cross-linked with 50 mL of 1% calcium chloride (CaCl_2_, 99%; CAS 10043–52–4, Synth, Diadema, SP, Brazil) under stirring to obtain a gel. Then, the gel was kept under refrigeration at 4 °C. To obtain the SPG skin dressing, the casting mold was assembled, with a square glass of 10.5 cm^2^ fixed at the bottom part and a round casting mold containing 19 circles of 2 cm diameter each in the upper part. Then, with a spatula, the gel was equally spread into the circles, and the casting was immersed in a second cross-linking of 2% CaCl_2_ for 20 min. The residues were washed out with distilled water, and every SPG skin dressing was removed from the casting method.

The skin dressings were placed in packages (five units per package), sealed, and frozen at −4 °C for 24 h. After freezing, the package was placed in a freeze-dryer and kept for 2 h. Subsequently, the SPG skin dressings were removed from the package, placed in a Petri dish, and left in a flow chamber with UV light turned on for 24 h (12 h each side) to sterilize them.

### 2.3 Material Characterization

For scanning electron microscopy (SEM) analysis, the SPG skin dressings were evaluated under a JEOL microscope (JSM-7500F). To perform that, the SPG skin dressings were coated with gold and mounted on an aluminum stub using carbon tape. Fourier-transform infrared (FTIR) spectra were analyzed using direct transmittance with KBr pellets in the range of 4000–500 cm^–1^.

### 2.4 In Vitro Studies

#### 2.4.1 Cell Lines

L929 and HFF-1 fibroblast (murine and human, respectively) cell lines were used in this study. They were, respectively, cultured in DMEM supplemented with 10 and 15% of bovine fetal serum (BFS) (Vitrocell, Campinas, SP, Brazil) and maintained at 37 °C in a humid atmosphere of 5% CO_2_ at subconfluence. For the in vitro analysis, the indirect method based on extracts from the skin dressings was obtained. For this purpose, 1 g of the SPG skin dressing was inserted in 50 mL of DMEM in a Falcon tube, followed by incubation at 37 °C and 5% CO_2_ for 24h and filtration (a 0.22 μm filter, Kasvi, Curitiba, Brazil). This solution resulted in an extract of 100%, and to obtain concentrations of 50 and 25%, DMEM was inserted in proportions of 1:1 and 1:3, respectively. Besides that, the control group (CG) was constituted by cells without any intervention, cultured in standard culture medium.

#### 2.4.2 Cell Viability (MTT)

The cell viability of the SPG skin dressing was evaluated through an MTT assay (3-(4,5-dimethylthiazol-2-yl)-2,5-diphenyltetrazolium bromide—Merck, St. Louis) in triplicate (*n* = 3). In a 96-well culture plate, a concentration of 1 × 10^3^ cells was seeded in 200 μL of DMEM. After 24 h, the media were removed and replaced with fresh DMEM in the CG and with 100, 50, or 25% extract in the treated groups. After 1, 3, and 7 days of incubation at 37 °C in a humidified incubator with 5% CO2, the media were taken out, and 50 μL of MTT solution was added to each well. To shield it from light, the plate was covered with aluminum foil and incubated for 3 h. Following the addition of 100 μL of isopropanol to lyse the cells, the plate was measured at 540 nm using a microplate spectrophotometer (BioTek Instruments, Inc.).

### 2.5 In Vivo Studies

#### 2.5.1 Experimental Groups

This study was performed following the Guiding Principles for the Use of Laboratory Animals after approval from the Animal Care Committee of the Federal University of São Paulo (3965170222).

Twenty-eight adult male Wistar rats (*Rattus norvegicus*), weighting ±350 g and 12 weeks old, were used. The animals were randomly divided into two groups and two time points (as described below in Table 1, *n* = 7 per group), with free access to commercial food and water, and kept in a room with a 12 h dark/light cycle at 24 ± 2 °C.

**Table 1:** Experimental Groups, Time Points, and Treatments of Animals

group	time point	treatment
control (CG)	5 and 10 days	skin wounded animals without treatment.
SPG dressings–treated (TG)	5 and 10 days	skin wounded animals treated with SPG dressings.

#### 2.5.2 Experimental Protocol

The animals were anesthetized via intraperitoneal injection with 80 mg/kg ketamine (Vetnil; Louveira, São Paulo, Brazil), 8 mg/kg xylazine (Syntec; Barueri, São Paulo, Brazil) and a single dose of 60 mg/kg Pentabiotic antibiotic (Zoetis; Brazil).

Subsequently, in a ventral decubitus position, their backs were trichotomized and sterilized for a surgical procedure. After that, a 15 mm circular dermatological punch was used to determine the site of the injury (at the interscapular region), and a full-thickness wound was performed using surgical scissors to remove the skin. SPG dressing was placed in the TG by suturing it at cranial, caudal, and bilateral points of the intact skin adjacent to the wound. All of the animals (CG and TG) were kept in heated environments to avoid hypothermia to recover from anesthesia and then relocated back to their cages.

The behavior of the animals was observed every day until euthanasia (5 or 10 days after surgery) via anesthetic overdose.

#### 2.5.3 Sample Collection and Preparation for analysis

After euthanasia, 4 cm^2^ in the wound area was removed. The collected site was determined with a transparent plastic mold. The tissues were placed in formalin solution, followed by a graded series of ethanol and xylol to dehydrated the samples. Next, the tissues were embedded in paraffin blocks, and histological sagittal serial sections (5 μm) were done using a micrometer (Leica Microsystems SP 1600, Nussloch, Germany). For histopathological and histomorphometry analyses, the slides were stained with hematoxylin and eosin (H.E. - Merck, Germany). Also, other sections were made for immunohistochemistry.

#### 2.5.4 Biological Evaluations

##### 2.5.4.1 Histopathological Analysis

Qualitative analysis of the wounds was performed using the histological sections previously prepared, which were evaluated under an optical microscope (Carl Zeiss, Oberkochen, Germany) at a magnification of 400×. Two evaluators (FO and FVBD) were blinded to the treatment, and an analysis was performed to evaluate the presence of inflammatory infiltrate, granulation tissue, reticular and papilar dermis, hair follicles, sebaceous glands, re-epithelialization, and the presence or absence of foreign bodies in the region of the wound.

##### 2.5.4.2 Histomorphometric Analysis

Photomicrographs of histological sections at 100× magnification from the CG and TG were taken. The images were analyzed in ImageJ 1.53e software to evaluate the following parameters:

##### 2.5.4.3 Neoepithelial Length

To evaluate the length of the epithelium, the measurement from the innermost hair root of the marginal skin to the end of the neoepithelium on each side of each cross-sectional area of all species at 100× magnification was considered. The sum of the lengths of the epithelium was evaluated for both sides.^30^

##### 2.5.4.4 Granulation Tissue Area

The fragmental structure of the newly produced dermis-like tissue and the transitional boundary layer of the continuous parallel structure of the dermis defined the side border of the regenerated area. The measurement did not include the upper epithelium since the lower border of the regenerated area was above the panniculus carnosus.^30^

##### 2.5.4.5 Collagen Birefringence

To distinguish between collagen fiber types I and III, histological sections were dyed with Sirius-red and examined under polarized light. An Axio Observer was used to take each photomicrograph using A D1 microscope with a 40× objective attached to a computerized imaging system using Axio Vision 4.8 Zeiss software (Zeiss, Thornwood, NY, USA). Nine blinded photomicrographs of the wound edge region were taken after sections were assessed.

The volume fraction (*V*_v_) quantification of collagen fiber types I and III was determined by a grid occupying an area of 10.000 μm^2^ containing 36 cross-shaped points overlapping Sirius-red photomicrographs.^131^ The volume occupied by each collagen fiber type was estimated by a single researcher counting the number of points hitting fiber types I or III as well as the sum of types. To obtain the *V*_v_ of type I or III collagen fibers, we determined the total number of points (*P*_t_) that coincided on the total analyzed region and the partial number of points on each tissue of interest (*P*_p_) and then calculated it using the formula *V*_v_ = Σ*P*_p_/Σ*P*_t_ (×100).^31^

##### 2.5.4.6 Immunohistochemistry Analysis

The fibroblast growth factor (FGF) and transforming growth factor β (TGF-β) were evaluated. For antigen retrieval, slides were submitted to rehydration in graded ethanol, followed by a pretreatement in a steamer with 0.01 mL of citric acid buffer (pH = 6) for three cycles at 850 W and 5 min each. Next, the material was preincubated with 0.3% hydrogen peroxide in PBS for 5 min and blocked with 5% normal goat serum in PBS for 10 min. Then, they were incubated overnight at 4 °C with anti-TGF-β and anti-FGF polyclonal primary antibodies (Santa Cruz Biotechnology, USA) and washed twice in PBS for 10 min. Subsequently, the sections were incubated with biotin-conjugated secondary antibody antirabbit IgG (Vector Laboratories, Burlingame, CA, USA) at a concentration of 1:200 in PBS for 1 h, washed twice, and applied avidin–biotin complex conjugated to peroxidase for 45 min. Bound complexes were performed with the application of a 0.05% solution of 3–3′-diaminobenzidine and counterstained with Harris Hematoxylin.

Digital images were captured at 200× magnification by an optical microscope, and the results were analyzed qualitatively (presence and location of the immunomarkers) and semiquantitatively, considering the percentage of the immunostained area evaluated according to the scale from 0 to 4 (0 = absent, 1 = <25%, 2 = 25–50%, 3 = 50–75%, and 4 = >75%) for immunohistochemical analysis.^32^

### 2.6 Statistical Analysis

The data were submitted to statistical analyses through distribution using the Shapiro–Wilk normality test. In the case of nonparametric data, the Kruskal–Wallis test and Dunn post hoc test were used, while parametric data were evaluated through one-way analysis of variance (ANOVA) and Tukey post hoc test. The software GraphPad Prism 6 (GraphPad Software, San Diego, CA, USA) was used, and the results were expressed as mean ± standard deviation (SD), considering differences of *p* ≤ 0.05 for demonstrating significance.

## 3 Results

### 3.1 Characterizations

**Figure 2:**
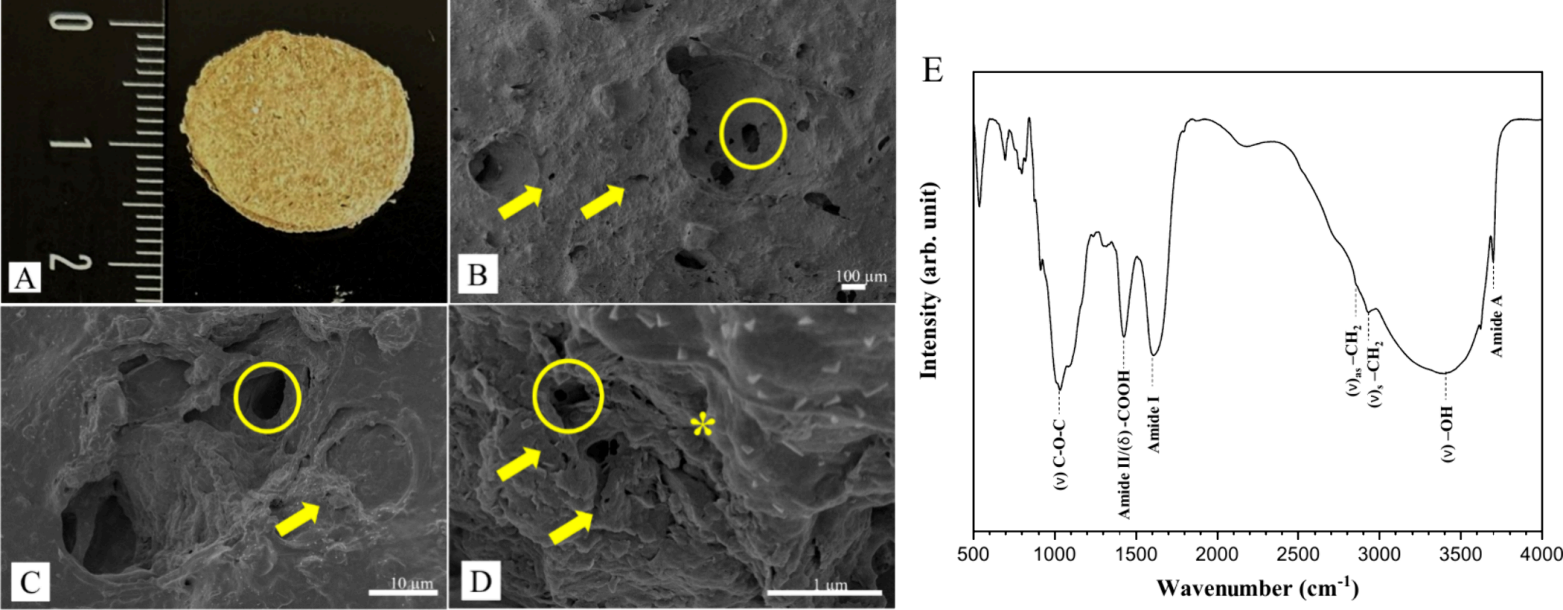
(A) Macroscopic and (B–D) SEM images from skin dressings (arrow represents micropore, circles represent macropore, and * represents the overlapping of SPG skin dressings). (E) FTIR spectra from SPG skin dressings.

Figure 2 represents the macroscopic image and SEM photomicrographs of the SPG skin dressing. The microscopic image of the SPG skin dressing is represented in Figure 2A (a diameter of 15 mm). It is possible to observe the round shape of the sample, with a rough surface and grayish color. Figure 2B,C demonstrates the SEM images of the skin dressings at magnitudes of 100 and 10 μm, respectively. In both micrographs, micropores and macropores could be seen along the samples. Moreover, the dressings present an irregular rough surface. Finally, at a lower magnitude (1 μm), it was possible to observe an overlapping of the SPG skin dressing layers (Figure 2D).

Figure 2E represents the spectra obtained from FTIR analysis for the SPG skin dressing. The FTIR peaks demonstrate the interaction of SPG and ALG, which are used in the composition of the SPG skin dressing. The peak at 1080–1030 cm^–1^ corresponds to C–O–C stretching, related to ALG, while 1429 cm^–1^ indicates Amide II, which is present in SPG but is also related to the bending of −COOH, which could be associated with ALG.^33^ Moreover, the peak between 1655 and 1620 cm^–1^ demonstrates the presence of Amide I, while the peaks at 2926 and 2858 cm^–1^ correspond to the asymmetric and symmetric stretching of −CH_2_ present in SPG and ALG, respectively.^34,35^ Finally, the peaks located at 3550 and 3240 cm^–1^ are related to Amide A, which indicates the N–H bonds common in collagen, along with the O–H stretching associated with ALG^36^.

### 3.2 Cell Viability

**Figure 3:**
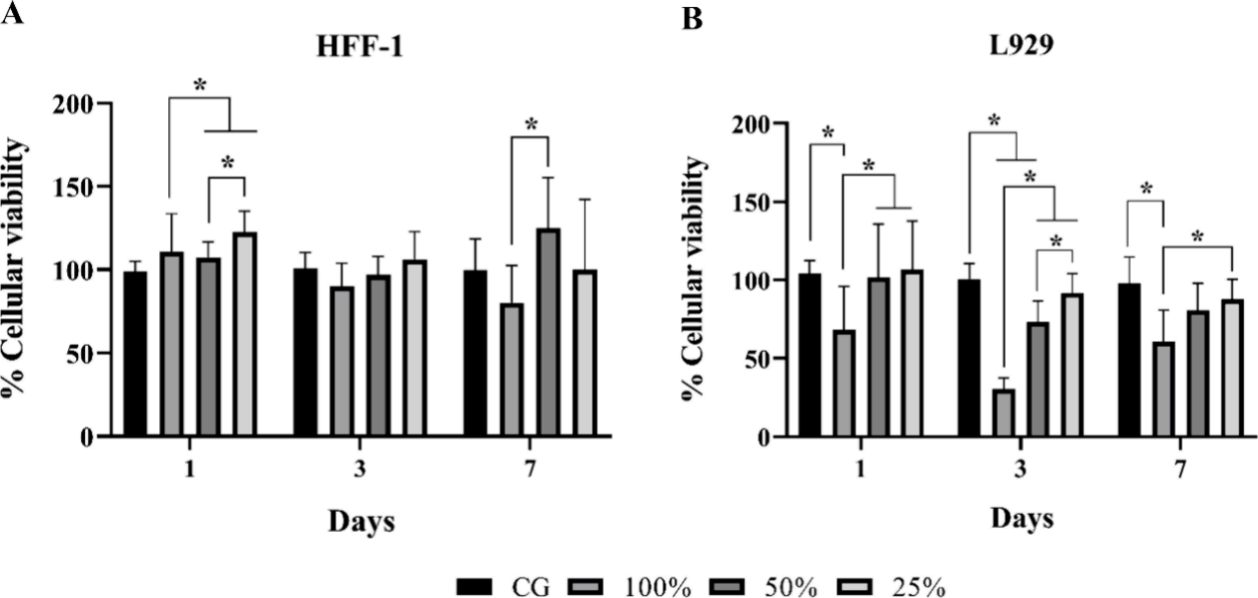
Cell viability of HFF-1 (A) and L929 (B) cell lines through MTT assay for SPG skin dressings incubated for 1, 3, and 7 days (mean ± SD, *n* = 13), *p < 0,05.

Figure 3 represents the cell viability of HFF-1 and L929 for SPG skin dressings.

For the HFF-1 cell line, on day 1, it was possible to observe that CG did not have a statistical difference compared to other experimental groups, while for the 100% extract, a statistically significant difference was observed compared to the 50 and 25% extracts. The 50% extract had a statistical difference compared to 25%, but the 25% extract did not demonstrate a statistical difference compared to other experimental groups. On day 3, no statistically significant differences were observed for any group. At the last experimental period (7 days), the CG, 50, and 25% extracts did not demonstrate a significant difference, but a statistically significant difference was observed for the 100% compared to the 50% extract.

Furthermore, the results for the L929 cell line demonstrated that the CG had statistically significant differences when compared to the 100% extract on day 1. Also, for the 100% extract, there was a significant difference compared to 50 and 25%, but the extracts 50 and 25% did not demonstrate a significant difference compared to other experimental groups. On day 3, there was a significant difference in the CG compared to the 100 and 50% extracts. Also, a difference could be observed for the 100% extract compared to 50 and 25%, and the 50% extract compared to 25%. Finally, on day 7, a significant difference was observed in the CG compared to the 50% extract, and 100% compared to 25%, but no difference was observed in the 50% extract.

### 3.3 Histological Analysis

**Figure 4:**
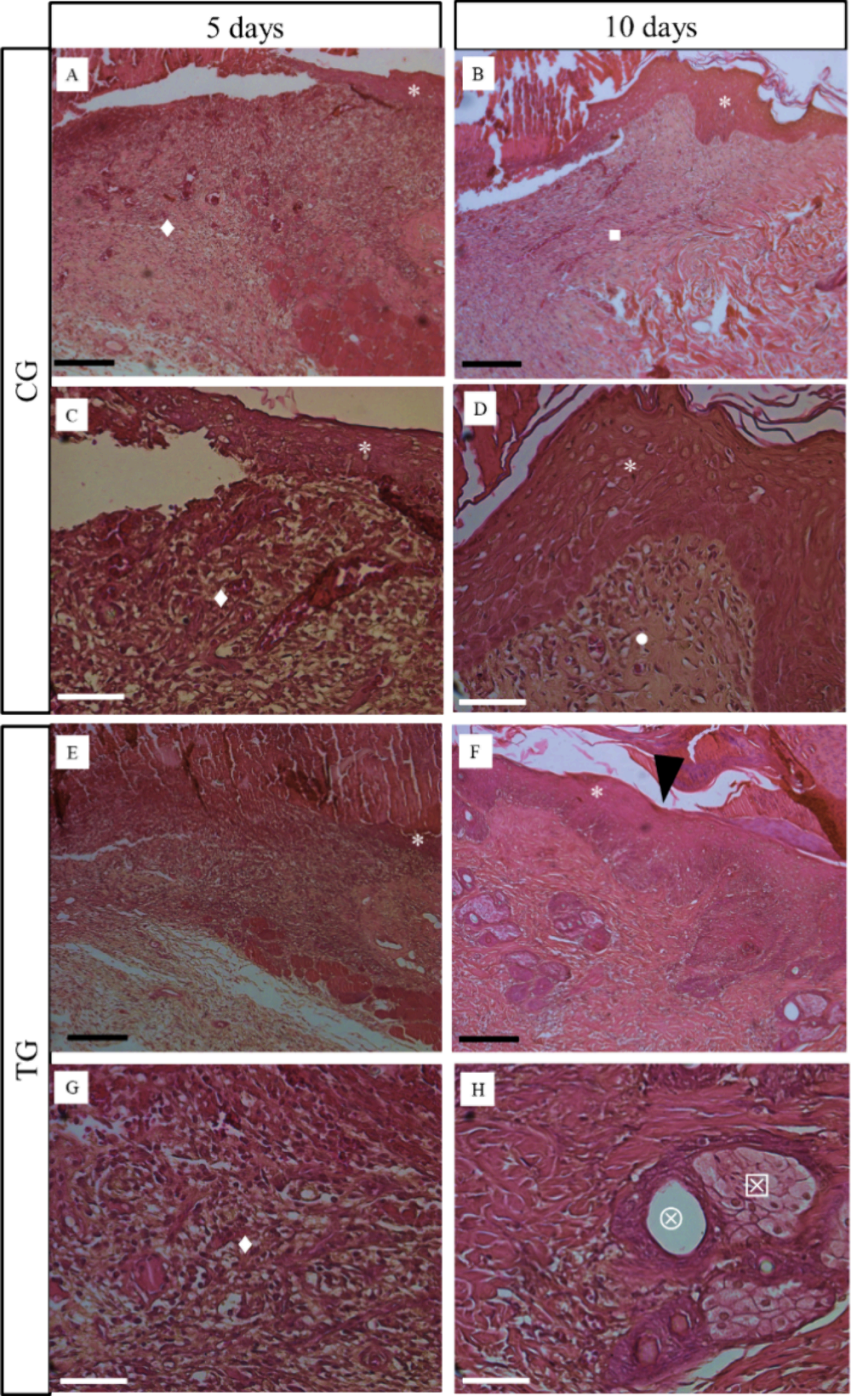
Representative images of histopathological aspects of skin wounds in rats of (A–D) CG and (E–H) TG followed for 5 and 10 days. *, epidermis; **◆**, inflammatory infiltrate; **■**, reticular dermis/granulation tissue; **•**, papillary dermis/granulation tissue; 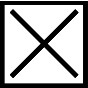, sebaceous gland; **⊗**, hair follicle. **Black head of arrows** indicates the re-epithelization process. Black scale bar = 200 μm; white scale bar = 50 μm.

Figure 4 demonstrates the histopathological analysis for the control group (CG) and treated group (TG), at 5 and 10 days postinjury. For the CG, after 5 days, at the site of the injury, an area without the presence of epidermis was observed; and in the region corresponding to the dermis, an intense inflammatory process was observed (Figure 4A). Figure 4C (higher magnification) demonstrates the presence of the inflammatory process with the presence of blood vessels. After 10 days of injury (image of 200 μm), it could be observed that the CG had a thicker epidermis at the border of the lesion. Also, the reticular dermis showed a transition with the presence of granulation tissue (Figure 4B). Figure 4D demonstrates a thicker epidermis, with the papillary dermis surrounded by granulation tissue.

Furthermore, for the TG (200 μm), at the experimental period of 5 days, the presence of the epidermis in the region of the wound (Figure 4E) and an intense inflammatory process around the region of the dermis at higher magnification were also observed (Figure 4G). At the experimental period of 10 days, a re-epithelialization process in the wound area was observed (Figure 4F). Moreover, sebaceous glands associated with hair follicles were observed (Figure 4H). For both scales, no foreign bodies were found in the analyzed regions.

#### 3.3.1 Histomorphometric Analysis

**Figure 5:**
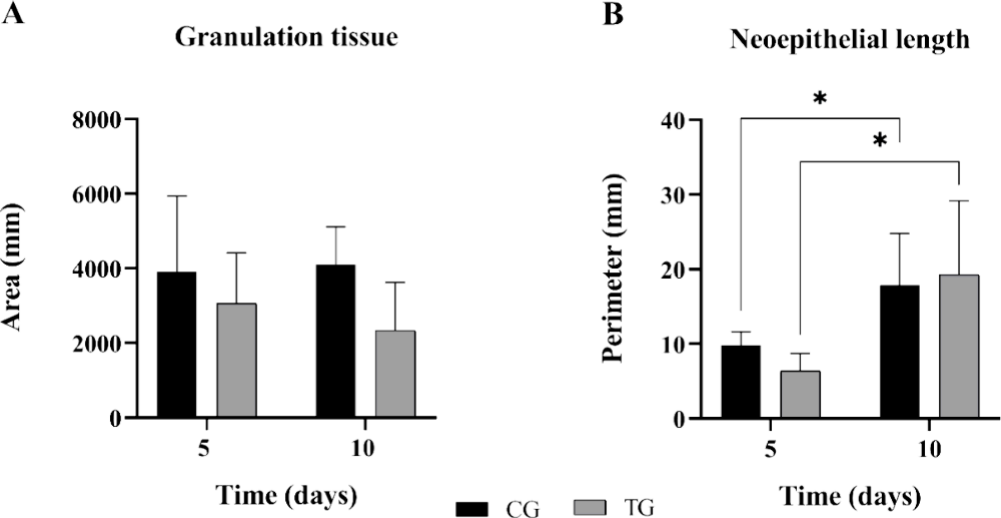
Representative histomorphometry results of (A) granulation tissue and (B) neoepithelial length (mm) evaluated at 5 and 10 days. Statistical difference (*p* = 0.0216).

Figure 5 shows the values found in the histomorphometry analysis for both groups in the experimental period of 5 and 10 days postsurgery. The values found for the area of granulation tissue for the CG were 3906.56 ± 2024.62 at 5 days and 4095.89 ± 1017.75 at 10 days (Figure 5A). In addition, for the TG, they were 3055.38 mm ± 1354.67 at 5 days and 2334.08 mm ± 1289.52 at 10 days. Moreover, the mean ± standard deviation (SD) results of neoepithelial length for the CG were 9.81 mm ± 1.85 at 5 days and 17.860 mm ± 6.93 at 10 days. Furthermore, for the TG, they were 6.38 mm ± 2.31 at 5 days and 19.29 mm ± 9.90 at 10 days (Figure 5B). Also, no statistical difference was found for granulation tissue, while for neoepithelial length, an intragroup difference was observed between the two experimental set points, although there was no intergroup difference (*p* = 0.0216).

### 3.4 Quantification of Collagen Fibers

#### 3.4.1 Collagen Fiber Analysis

**Figure 6:**
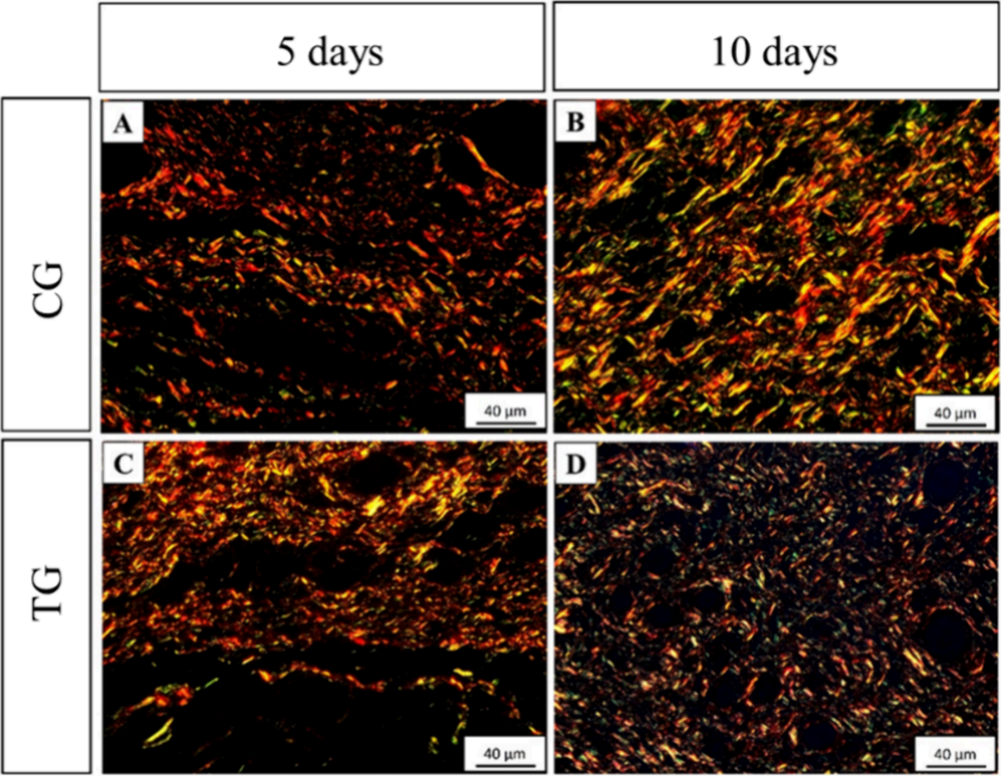
Representative photomicrographs of reticular dermis collagen fibers of the CG and TG groups 5 or 10 days postinjury. Stained with Sirius-red and analyzed under polarized light, the images show the differentiation between type I (red, orange, and yellow) and type III (green) collagen fibers.

Photomicrographs of reticular dermis collagen fibers were obtained and showed organized in meshes arranged in a network pattern, presenting type I and type III collagen fibers in all groups (Figure 6). The groups evaluated 5 days post injury (Figure 6A,C) presented thicker connective tissue fibers arranged in a connective tissue network. The groups evaluated 10 days after injury presented a closed connective tissue network. In addition, the TG at day 10 exhibited thin collagen fibers in a closed tissue network when compared with the CG evaluated in the same period (Figure 6B,D).

**Figure 7:**
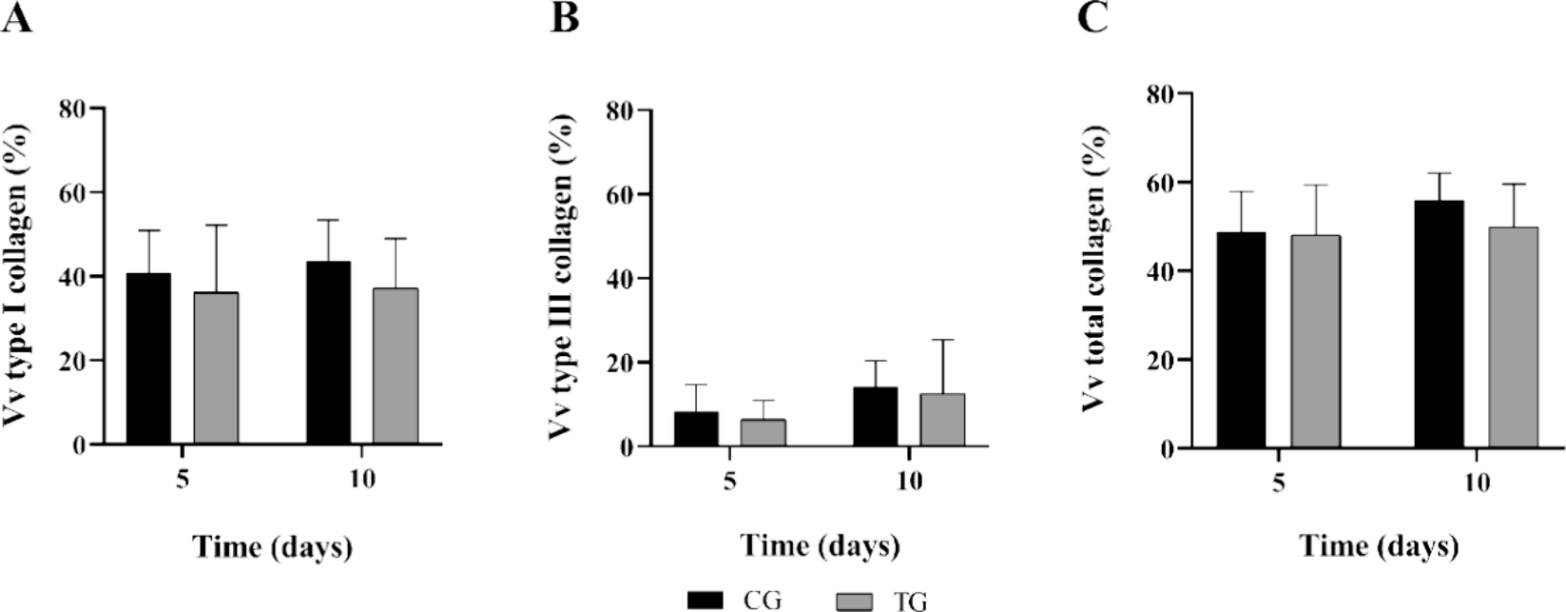
Representative birefringence of the control (CG) and SPG-treated (TG) groups. Volume fraction (Vv) in percentage of (A) type I, (B) type III, and (C) total collagen, 5 or 10 days postinjury.

Figure 7 shows the morphometric evaluation of the volume of collagen fibers, differentiating them into type I collagen, type III collagen, and total collagen. There were no statistical differences between the groups or time points for the different types of collagen evaluated.

### 3.5 Immunohistochemistry Analysis

**Figure 8:**
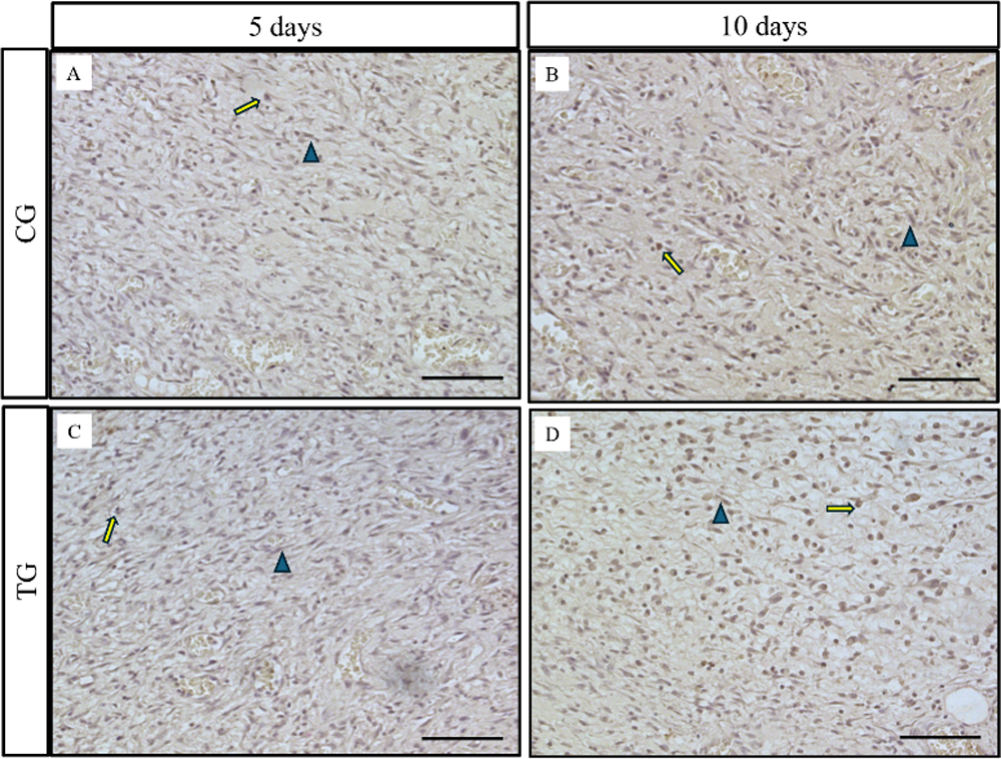
Representative photomicrographs of TGF-β immunohistochemical analysis for 5 and 10 days (yellow rightward arrow (fibroblast) and blue triangle (macrophage). Scale: 50 μm).

**Figure 9:**
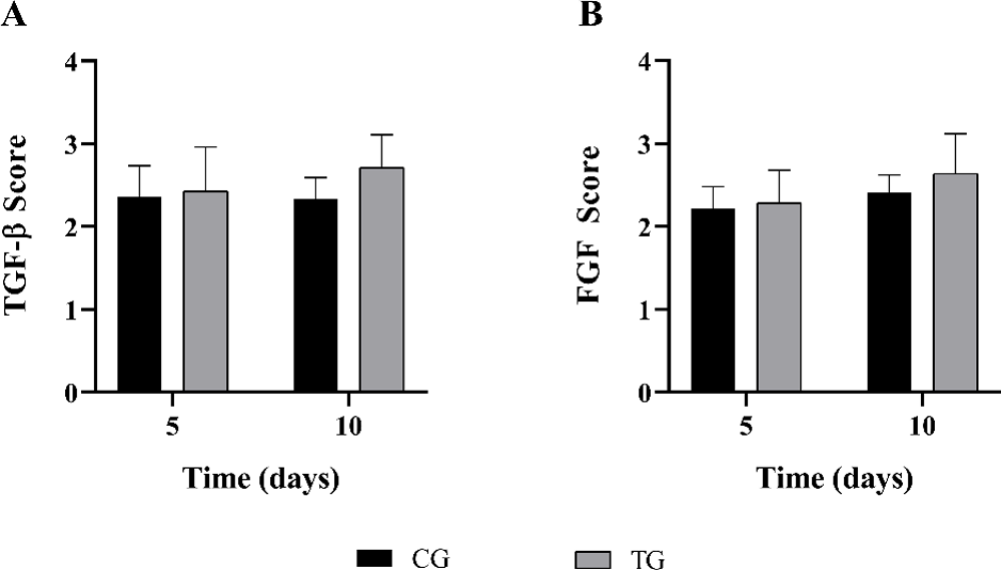
Representative results of semiquantitative immunohistochemistry evaluations of (A) TGF-β and (B) FGF scores evaluated for 5 and 10 days.

**Figure 10:**
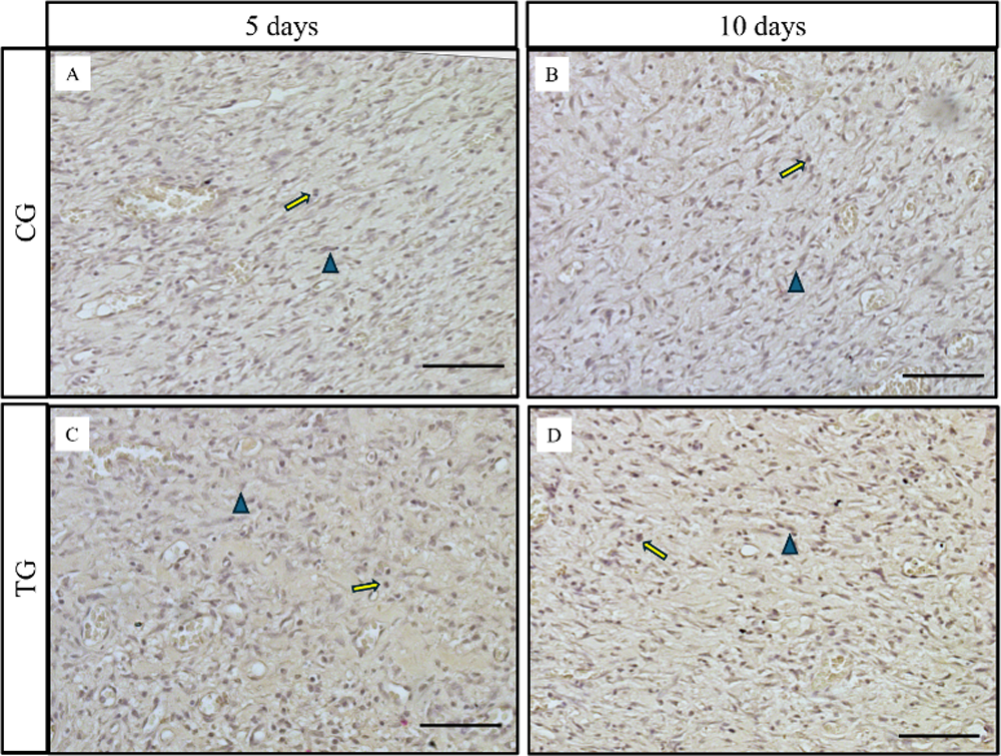
Representative photomicrographs of FGF immunohistochemical analysis for 5 and 10 days (fibroblast and macrophage. Scale: 50 μm).

Figure 8 illustrates the immunostaining of TGF-β, mainly in fibroblasts in both the CG and TG over 5 and 10 days. In the semiquantitative analysis (Figure 9), no differences were observed either between the groups or between the time points evaluated (*p* = 0.1955 and 0.4039, respectively). Figure 10 shows the immunostaining of FGF in fibroblasts and endothelial cells in both the CG and TG at the different times analyzed. In relation to the semiquantitative analysis (Figure 9), no differences were found between the groups or the experimental periods (*p* = 0.0536 and 0.2904 respectively).

## 4 Discussion

The aim of the present study was to evaluate the effects of an innovative skin dressing for treating skin wounds using an experimental model in rats. Initially, the characterization techniques employed in this work confirmed that the wound dressing possesses a porous structure and retains the composition of ALG and SPG after the manufacturing process. Furthermore, in vitro assays demonstrated the absence of cytotoxicity in the samples when they were cultured with murine (L929) and human (HFF-1) fibroblast cell lines. The histological and histomorphometric evaluations demonstrated similar results among the experimental groups, with no signs of dressing rejection or exacerbated inflammatory processes at the site of injury in the treated animals. The picrosirius analysis demonstrated that both type I and type III collagen fibers were seen for all groups. Immunohistochemistry analysis showed similar patterns of staining for all immunomarkers.

Collagen-based skin dressings have been widely utilized to promote tissue healing in burns and wounds, according to data from literatures.^19,37^ Because of its bioactivity and suitable mechanical qualities, collagen is one of the most crucial biomaterials to be used in tissue engineering suggestions.^38^ In this regard, scientists were inspired to conduct the current study due to the continuous need for novel collagen sources.

The porous structure observed in the SEM analysis of the manufactured dressings represents a significant advantage for skin regeneration. Porosity plays a crucial role in wound healing by allowing the absorption of wound fluids and facilitating oxygen supply to the affected area.^39^ The casting technique followed by freeze-drying, known for its ability to create macroporous structures, provides an excellent approach for fabricating such dressings. This technique not only enables precise control over the porosity of the material but also encourages cell growth and integration with the surrounding tissue, essential for successful regeneration.^40^ The interconnected pores created during casting promote the exchange of gases and nutrients, which are vital for the healing process.^41^ Additionally, casting is a cost-effective and straightforward method, making it accessible for producing biocompatible materials like SPG and ALG.^11^ Similar findings were reported by Sales et al.,^29^ who used the casting technique to develop wound dressings from marine sponges. Their study also demonstrated a macroporous structure with interconnected fibers, offering suitable mechanical strength. Furthermore, FTIR analysis confirmed that the casting process preserved the integrity of both the ALG and SPG structures, which is crucial for supporting the regeneration process.

The in vitro studies demonstrated positive results from the MTT analysis for the proliferation of both cells (except for 100% compared to the CG on day 3 for L929) in the different groups evaluated. Sousa et al.^42^ demonstrated that collagen from a marine species also did not demonstrate genotoxicity in in vitro studies using fibroblasts, suggesting that this material was biocompatible and noncytotoxic, being regarded as an appropriate substance for tissue engineering concepts. It is important to note that according to ISO 109333–5:2009, a substance intended for use in medical applications may be deemed harmful to cells if it causes more than 30% of deaths. In the current study, both HFF-1 and L929 showed at least 70% cell viability for the extract groups as compared to the CG (with the exception of L929 at day 3 for the 50% extract). Given that collagen is a component of the extracellular matrix of many tissues and can promote cell attachment and growth, these studies demonstrate the beneficial effects of marine collagen on promoting cell metabolism and proliferation.

The qualitative histological analysis showed that the results obtained for every experimental group were comparable to and consistent with those obtained during the proliferative stage of tissue restoration. It is important to highlight that no signs of rejection, necrosis, or exacerbated inflammatory processes were seen in the treated groups, which is a very positive sign indicating the biocompatibility of the skin dressings. Foreign bodies are a common response to the presence of biomaterials that have been implanted; they are created by the fusion of macrophages, which are usually found at or close to the interface between the biomaterial and the host tissue. Their presence is related to the index of biocompatibility.^43^ In addition, another sign of biocompatibility is the presence of an exacerbated inflammatory process after the graft is implanted.^44^ These statements suggest that the marine collagen skin dressings are a noncytotoxic, biocompatible substance that will likely be reabsorbed over time and replaced by newly created tissue. Sales et al.^29^ demonstrated that skin dressings manufactured from marine sponge collagen presented adequate physical characteristics and demonstrated biocompatibility in an experimental study using a wound healing animal model. Also, it is worthwhile to emphasize that the similar results found in experimental groups may also be related to the use of a noncritical skin wound model. The process of regeneration occurs naturally, even though it is crucial to assess the impact of interventions to speed up the healing process using noncritical injury models. Because of this, the outcomes of the control group are sometimes comparable to or superior to those of the treatment groups, particularly when biomaterials are utilized.^45^ However, the histological findings in the treated group can be faced as positive results, especially related to biocompatibility.

Related to collagen analysis, treated groups presented a more closed tissue network, indicating a more mature aspect of collagen deposition. It is well-established that collagen constitutes a class of proteins found in connective tissues’ extracellular matrix that help preserve bone tissue’s structural integrity. At the commencement of mineral deposition, type I collagen mainly offers structural and mechanical support, serving as a natural scaffold for other extracellular matrix proteins outside of their core paper.^46^

During the normal process of wound healing, multiple growth factors such as TGF-β and FGF are expressed, aiming to activate a cascade of steps to induce neoangiogenesis, re-epithelialization, and tissue healing.^47^ Interestingly, the immunohistochemical expression for both immunomarkers demonstrated a similar pattern. TGF-β is known a multifunctional cytokine involved in large number of cellular functions, stimulating fibroblast migration and proliferation, collagen production, and extracellular matrix (ECM) deposition in the wound healing process^48,49^. The study of Elbialy et al.^50^ in an experimental model used to examine the effects of tilapia skin collagen extract on the healing process of skin wounds showed increased TGF-β immunostaining 15 days after surgery, particularly in fibroblastic cells. It is possible that the skin dressing’s stimulation was insufficient to activate the intracellular signaling pathways that raise the expression of this growth factor.

Furthermore, FGF is essential for many biological processes, such as tissue regeneration and repair, cell proliferation, survival, metabolism, morphogenesis, and differentiation.^48^ Also, no difference was seen in the expression of FGF in the treated groups. The same hypothesis can be raised, stating that the stimulus offered by the treatment was not able to produce molecular modifications culminating in the observed increase in FGF immunostaining.

The highlights of the present study are the observation of the biocompatibility of the skin dressing manufactured with marine collagen and the more mature aspects found for collagen deposition in the treated animals. In vitro investigations utilizing fibroblasts and osteoblasts have shown that collagen from marine sponges isolated from the species *Aplysina fulva* is not cytotoxic.^51^ Furthermore, fibroblast cells were not cytotoxically affected by *Chondrilla caribensis* collagen extract.^18^ Marine collagen from sponges is biocompatible and promotes cell survival, as demonstrated by Silva et al.^15^ This study showed that the marine collagen dressing may integrate with the tissue in this situation without exacerbating the inflammatory process or causing tissue rejection. However, for further studies, critical size models should be used to better evaluate the effects of skin dressings.

## 5 Conclusions

Therefore, the current investigation showed that SPG skin dressings exhibited in vitro biocompatibility and improved re-epithelialization in the in vivo experimental model, with no signs of rejection or exacerbated inflammatory processes. These findings point out the promising biological effects of the new skin dressing based on SPG marine collagen, indicating that this could be an alternative therapeutic intervention for treating wounds, corroborating the initial hypothesis. However, further studies using a critical wound healing injury model should be used, associated with longer experimental periods of evaluation, to further investigate the effects of these promising therapeutic approaches throughout the skin repair process.

## Abbreviations

SPG: spongin
SC: spongin-like collagen
CG: control group
TG: treated group
SD: standard deviation
Vv: volume fraction
TGF-β: transforming growth factor beta
FGF: fibroblast growth factor
